# Stakeholder-driven, consensus development methods to design an ethical framework and guidelines for engaged research

**DOI:** 10.1371/journal.pone.0199451

**Published:** 2018-06-21

**Authors:** Giselle Corbie-Smith, Mysha Wynn, Alan Richmond, Stuart Rennie, Melissa Green, Stephanie M. Hoover, Sable Watson-Hopper, Kyle Simone Nisbeth

**Affiliations:** 1 Department of Medicine, University of North Carolina School of Medicine, Chapel Hill, North Carolina, United States of America; 2 Department of Social Medicine, University of North Carolina School of Medicine, Chapel Hill, North Carolina, United States of America; 3 Project Momentum, Inc., Rocky Mount, North Carolina, United States of America; 4 Community-Campus Partnerships for Health, Raleigh, North Carolina, United States of America; University of Liverpool, UNITED KINGDOM

## Abstract

Increasingly, researchers seek to engage communities, patients, and stakeholders as partners in the process and products of health research. However, there is no existing stakeholder-driven ethical framework for such engaged scholarship. We employed an iterative, stakeholder-engaged method to develop a data-driven framework for the ethical review and conduct of engaged scholarship. We used consensus development conference methods and a modified Delphi survey to engage 240 community members, ethicists, and academic researchers. This multi-staged process produced a framework with 4 domains: vision of equitable and just research, relationship dynamics, community-informed risk/benefits assessment, and accountability. Within the framework, 4 cross-cutting considerations and 15 statements explicate the stakeholders’ priorities for the ethical review and conduct of engaged scholarship. Though the findings are promising, the study is limited in that it focuses on stakeholder perspectives, but does not actually evaluate or apply the findings in the field. The stakeholder-engaged framework provides a platform for further articulation of ethical practices and policy for engaged scholarship.

## Introduction

In both developed and ascending nations, there is growing interest in engaging communities, patients, and stakeholders as partners in the process and products of engaged scholarship [1]. Engaged scholarship is an umbrella term that encompasses a diversity of existing nomenclature, including patient-centered outcomes research, community-based research, and community-based participatory research [2]. In biomedical and public health research specifically, engaged scholarship has been traced back to Kurt Lewin’s demonstration projects in the 1930s [3]. Present day, engaged scholarship emerges as a novel approach to translational and dissemination research [4].

Engaged scholarship in public health and biomedical research is grounded in an *ethos* of social justice and a common goal: to address the health concerns of populations living in underserved, economically constrained, or minority communities [2]. We use the word community broadly, to capture a non-homogenous group of people with diverse characteristics who are linked by social ties, share common perspectives, illness, or health conditions, and who engage in joint action to advance health [5]. To achieve health equity, engaged scholarship assumes that knowledge is co-owned and co-created within communities. Engaged scholarship flattens conventional power and knowledge hierarchies between researcher and participant by sharing power and decision-making. In contrast to being relegated to the role of “research subjects” or being rendered invisible in the academic’s research agenda, nonacademic partners offer essential expertise to solve complex problems in public health and health care [2].

With a shared commitment to social justice and action, engaged scholarship encourages ethical research practices [2, 6]. In terms of the ethical principal of beneficence, research questions in engaged scholarship often are responsive to social, economic, and political contexts that are associated with diminished power and autonomy of certain populations [7]. In research that uses engaged approaches, there is an expectation among academic and nonacademic collaborators that research will be designed to address inequities experienced by potential participants [8]. When determining potential participants, scholars refrain from practices such as targeting groups unfairly, excluding certain groups from participating, or by assuming that certain populations will be less likely to access research benefits [6].

The unique features of engaged scholarship give rise to an alternative set of tensions, conflicts, and dilemmas. Ethical issues in engaged scholarship include, but are not limited to, historical considerations of past scientific misconduct, socioeconomic inequities influencing incentive structures, and balance of competing interests of group or individual harms or benefits in communities that endorse a more collective ethos [6]. Turning to the existing literature, we show the limitations in the current evidence for the ethical review and conduct of engaged scholarship.

## Background

The Belmont Report [9] and the set of principles derived from it, has given researchers a tidy way of examining and resolving ethical conflicts as they relate to human subjects research. Analyses that engage the ethical principles of respect, beneficence, and justice, as well as the frameworks that derive from them, were developed for traditional research contexts where investigator-initiated research is reviewed by institutional review boards (IRBs) at academic institutions, conducted in controlled settings, and where communities play a more passive role [10]. A principle-based analysis of research ethics concerns prioritizes investigator expertise and scientific validity as primary criteria in the design and review of research [10]. Though investigator expertise and scientific validity are important in all research, additional considerations and expertise are needed to make ethical decisions in engaged scholarship [4].

Further, current practice and recent revisions to the Common Rule are intended to guide the review of human subjects research. However, the Common Rule as written and in practice may not cover the full range of ethical considerations that arise in engaged research approaches nor adequately address the needs and interests of community investigators [11–13, 6]. To ensure a review of the ethical concerns central to engaged research, some authors have suggested changes or additional layers to IRB review [11, 14, 15]. Many processes have been proposed: stronger community representation on IRBs; community-level considerations in policies, applications, and processes; increased understanding of engaged scholarship by the IRB; greater transparency of IRB deliberations and comments and an established community-level review, i.e. a blended review. Some recommendations have been implemented, for example by the Bronx Community IRB [16].

In addition to the necessary revisions to federal regulations and IRB processes, the field is so new that no comprehensive, data-informed, and stakeholder-driven examination of these issues has been undertaken; thus no clear recommendations exist. Attempts to address ethics have been primarily conceptual in nature. For example, Chen et al.’s framework is a specific application of an existing ethical framework for clinical research [11]. Hébert and colleagues’ ethical assertions are an extension of the principles of community-based participatory research [14]. Another gap in this literature is due to an overrepresentation, in that much of what has been published comes from the perspective of investigators, IRB members, or ethicists. A key exception is Ross et al.’s framework, which was developed with academic and community partners, as well as human subjects protection personnel [6]. Their framework highlighted issues with human subjects specifically, which is only one of many ethical considerations in engaged research.

As engaged scholarship promotes the engagement of patients, families, and community members as co-constructors of knowledge, community and academic partners require research ethics guidance that is appropriately matched to the complex ethical issues that arise in community-academic partnerships. The engagement of nonacademic partners and stakeholders is key to explicating research ethics for engaged scholarship. Our paper seeks to fill this gap in the literature with an author team of academic-community partners who engaged stakeholders in order to articulate an ethics framework.

Our purpose was (1) to engage a diverse group of community, ethics, and academic experts with a range of experience in engaged scholarship, which would (2) yield a stakeholder-engaged, data-driven framework for the ethical review and conduct of engaged scholarship. In this paper, we report on the iterative, multi-staged, stakeholder-engaged methods that we used in order to draw on the experience and expertise of community members/advocates, academic, bioethicists, and research ethics stakeholders. We provide the stakeholder-engaged framework and ethical recommendations to be applied to the conduct and review of engaged scholarship.

## Methods

### Multi-staged procedures

We used well-described consensus development methods, which are frequently applied to create clinical guidelines for a particular disease, clinical scenario, or discipline [17, 18]. By definition, consensus development methods are standardized approaches intended to organize the available evidence and opinions of experts and systematically convert them into guidelines that aim to improve practice and influence policy [19]. These methods often rely on both the opinions of experts and a literature review. The literature review is conducted to ensure all participants have a similar level of understanding and to highlight available evidence (or lack thereof) upon which the guidelines can be built. In our project, the experts were defined as community, ethics, and academic professionals with a range of experience in health-related engaged scholarship. The crux of expert engagement was via two consensus development procedures: consensus development conference methods and modified Delphi technique [19–21]. Consensus development conference methods require in-person interaction to facilitate dialogue, debate, and discussion of priority issues [15]. The modified Delphi ranking method employs rating and ranking of the priority of ethical statements using online surveys.

Consistent with consensus development procedures, we used sequential, iterative stages (see Table 1). In Stage 1, we prepared for stakeholder engagement by gathering expert opinion and evidence by (1) conducting a narrative literature review on engaged scholarship ethics and (2) soliciting case studies from experts [19]. In Stage 2, we engaged stakeholders via consensus development workshop and key informant interviews. The consensus development workshop was the primary data source, and informant interviews were conducted to achieve theoretical saturation [22], ensuring that the themes from the workshop were complete and representative of three distinct stakeholder perspectives: IRB, academic institutions, and community organizations. We synthesized the data from the workshop and interviews yielding 15 statements and 4 domains. In Stage 3, we sought a broader range of stakeholders by disseminating n modified Delphi survey online, which allowed us to finalize the framework. The survey was modified in the sense that we only completed a single data collection timepoint, whereas traditional Delphi methods call for multiple rounds of data collection to result in convergence. Because the results found an overwhelming endorsement of the 15 statements, convergence was achieved, and subsequent Delphi survey timepoints were not needed. All procedures were consistent with University of North Carolina IRB approval, which determined exempt status and waived informed consent procedures.

**Table 1:** Multi-staged methods.

Procedure & Purpose	Stakeholders Recruited and Participated	Collection Protocol	Analysis Protocol	Final Product
**Stage 1: Preparation for Stakeholder Engagement**				
Narrative literature review of engaged scholarship ethics articles to develop draft of ethical responsibilities	Analysis conducted by all author team members with academic and community affiliations.	1. Team member retrieved 804 unique articles from PubMed and Scopus. Search terms: (community-based participatory research, participatory research, community based research, consumer driven and research, OR community engaged research) AND (ethics OR morals). Required English language. 2. Team member completed initial screening using Covidence [23]. Exclusion criteria: Animal or biological studies, safety of field research, individual or emotion-based research. 652 articles were removed after title and abstract screening. 108 articles were removed after full text review, leaving 44 relevant articles.	1. Team member reviewed and re-reviewed articles to develop list of themes represented in any of the 44 articles. 2. Entire team developed initial draft of statements, reviewed statements, and revised statements.	12 recommendations on the ethical responsibilities for academic and community partners
Solicit case studies to be presented at the consensus development workshop	1. Team recruited from 500 health-related engaged scholarship experts, which included authors identified in the narrative literature review, registered attendees at a national conference on engaged scholarship, Community-Campus Partnerships for Health members, community engagement and ethics cores of the Clinical and Translational Science Award Consortium. 2. Of 500 individuals contact, 22 expressed interest and then 8 ultimately submitted case studies.	1. Author team requested 5–6 PowerPoint slides on the academic-community partnership, ethical challenges, relevance to engaged research, problem identification, and lessons learned 2. Of 8 received, 5 were from academic experts and 3 were from community experts. Case studies addressed: community regulatory review process, trust, miscommunication, informed consent, incentives, and duty to report suspected child abuse. .	1. Team selected subset of 4 case studies to represent 2 community experts and 2 academic experts. Presented cases were limited 4 to limit time on case review and increase time for consensus development procedures at the workshop.	4 case studies, 2 provided by community experts and 2 provided by academic experts
**Stage 2: Consensus Development with Stakeholders**				
Consensus development workshop to generate domain and revised ethical recommendations	1. Invited experts who submitted a case study. 2. 11 stakeholders participated in 1.5 day long workshop	1. 4 experts presented respective case studies. Team presented 12 statements. 2. Team facilitated small and large group sessions to discuss, refine, operationalize, rank recommendations and develop domains. Procedures were consistent with Consensus Development Workshop procedures [20]. 3. Team member audio recorded all workshop activities and maintained field notes of content and nonverbal observations.	1. Team reviewed workshop data to confirm that all data were captured in 15 statements and domains that were developed by the end of the workshop.	15 recommendations and 4 domains
Theoretical sampling interviews to ensure workshop data was saturated	Team recruited 2 IRB members from academic institutions, 2 community investigators, and 2 academic investigators.	1. Trained team member (Black woman research staff, MPH) completed individual interviews with semi-structured protocol outlining specified topics: community-engaged research experience, community-engaged research ethical considerations and recommendations. The protocol was developed from a grounded theory approach to build theory from the data gathered; specifically, the prompts addressed topics that required further analytical exploration from the consensus development workshop. 2. All participants completed interview and did not dropout. Interviews occurred in a private space in a public setting. They were audio recorded and transcribed (*M* = 38.5 minutes).	1. Team reviewed interview data together to ensure saturation and that no new themes emerged beyond what was established in the workshop. 2. Team reduced redundancies in statements and domains and grouped the 15 statements into the 4 domains.	15 revised recommendations and 4 domains
**Stage 3: Broader Stakeholder Engagement and Final Analysis**				
Modified Delphi survey to finalize recommendations and framework	1. Team recruited stakeholders from the narrative literature review, network of Community-Campus Partnerships for Health members, Community Based Public Health Caucus and National Community Based Organization Network, community engagement and ethics cores of the Clinical and Translational Science Award Consortium, other organizations known by the author team, and NIH Reporter system, as well as snowball sampling. 2. 240 stakeholders completed online survey.	1. Participants ranked all statements in order of importance. 2. Participants rated each statement’s importance on a five-point scale 3. Participants reviewed the statements and comment on challenges or recommendations they feel were not reflected in the list of recommendations.	1. Team conducted descriptive statistics and found high endorsement of ratings mean range of 4.32–4.75 where 5 is the highest level of endorsement. 2. Team conducted Student-Newman-Keuls (SNK) analysis, which found no statistically significant differences in the ranking of statements (Mean ranking = 6.65–8.88). Thus, there was no quantitative findings to support grouping any statements. 4. Team reviewed quantitative findings together and determined there was no basis to support grouping any statements, removing any statements, or otherwise revising statements. Team reviewed the open-ended responses and compared to existing recommendations and domains and made minor edits to ensure that all participant responses were represented in the final results.	Final 15 recommendations and framework.

The rigor of the study was ensured by multiple aspects of the design. First, multiple data sources were triangulated across the stages of data collection. In addition to data collection, the author team, which included both community and university affiliated researchers, sought consensus when analyzing data and interpreting findings. Data collection and analysis were both iterative processes, ensuring the trustworthiness of the final analysis.

## Results and discussion

### Engaged scholarship ethics framework

**Fig 1:**
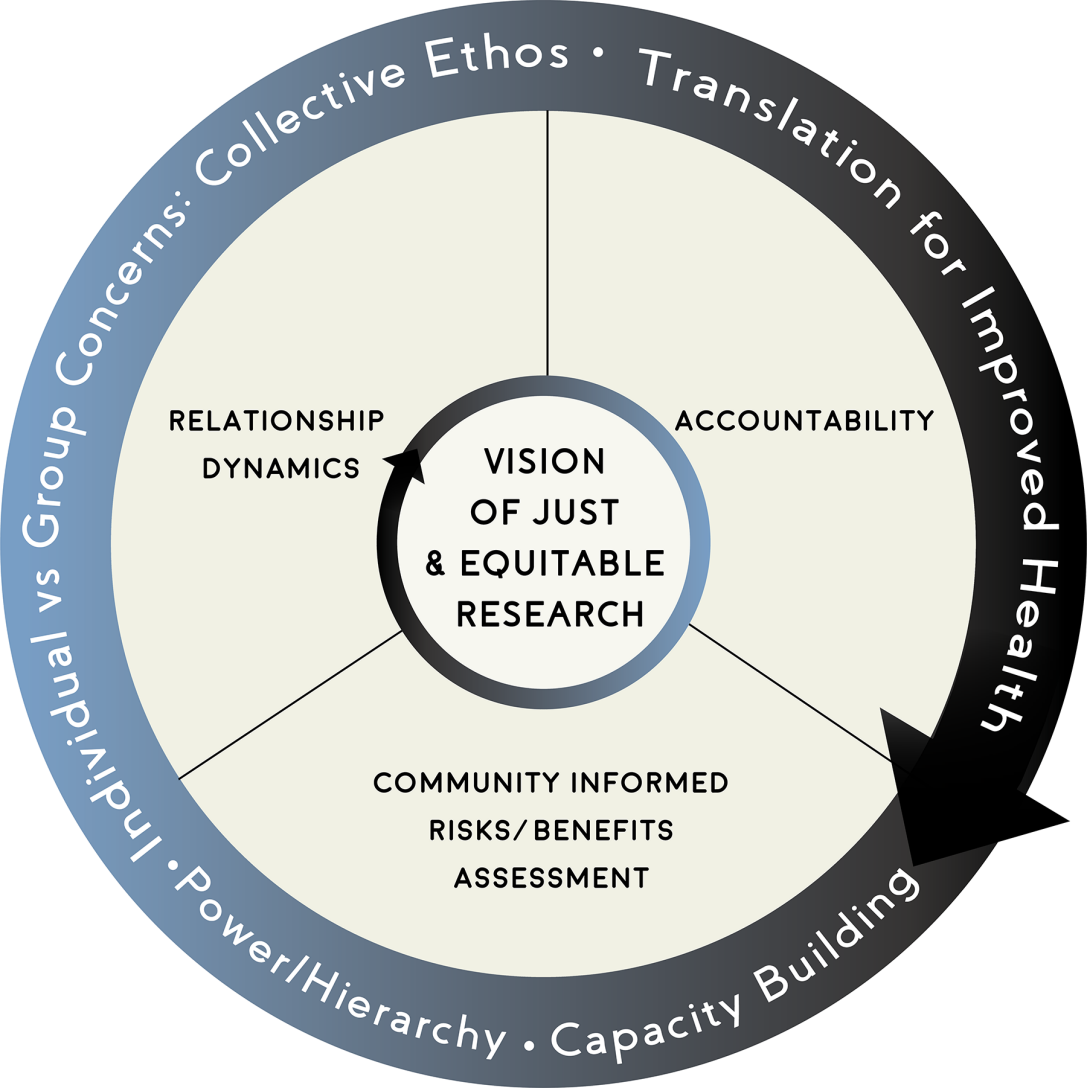
Engaged scholarship ethics framework. Note. Vision of just and equitable research is the overarching domain with three additional domains: relationship dynamics, accountability, and community-informed risks/benefits assessment. The outer circle represents the four cross-cutting considerations: capacity building, translation for improved health, power/hierarchy, and individual vs. group concerns: collective ethos.

The stakeholder-engaged, iterative analysis yielded an ethics framework for the review and conduct of engaged scholarship. The framework is anchored by four domains: vision of equitable and just research, relationship dynamics, community-informed risks/benefits assessment, and accountability. The four domains are further articulated by four cross-cutting considerations: capacity building, translation for improved health, individual vs. group concerns: collective ethos, and power/hierarchy (see Fig 1). Lastly, the 15 responsibility statements are nested within the four domains (see Table 2).

**Table 2:** Framework domains, illustrative quotes, and recommendation statements.

Domain	Illustrative quote	Recommendation Statement
Vision of Equitable and Just Research	Community member: “I see a community as … having the resources, and the people, and the energy, and the imagination, and the curiosity, and the strength to solve their problem … They’re looking for allies to help them through whatever process they have to go through in order to help level the playing field, and research is one of those [processes]. … [E]ngaged research makes it so much better for everybody, for the community to have the need and the dream to better themselves and for researchers and institutions that have so much in terms of knowledge and access to understand what’s going on in the community and to work as allies with communities.	1. Researchers and communities strive for active partnerships that honor shared power and resources, co-learning and mutual respect.
		2. Community engaged research is responsive to the structural conditions responsible for poor health and deprivation, and contributes to the improvement of fundamental participant and community welfare.
		3. Community engagement should be guided by a broad conception of justice.
		4. Community and academic researchers in partnership, determine whether and how proposed research is important, relevant, and valuable.
Relationship Dynamics	Community member: “I think when you’re going to engage in a community, you have to look at what their core values are. It’s not the same; every community is different. When you say community, what are you talking about? …Are you talking about Whites? Asians? Vietnamese? Thai? Hispanic? …. You’re not going to learn that by Googling. You need to get in there. You need to get there and engage, actually meet people and connect with people who are probably a part of that community … Start talking. Just start talking to them! That’s prior to you developing your structure because you need to have that relationship and that understanding of who they are and what their core values are.”	5. Those parties involved in community engaged research (CEnR) should engage the community of interest in the planning, implementation and dissemination of research.
		6. Researchers and communities should have transparent communication with one another to foster trustworthiness.
		7. Research should be initiated after first gaining familiarity with the setting in which the research will be conducted.
Community Informed Risk/Benefit Assessment	IRB member: “If we’re talking about sharing information about or disclosing the risk and benefits through the informed consent process, that’s one thing, …but then how can we expand that? I think by virtue of including the community earlier on in the research process, getting their input to that process, so that the research questions, research methodology, the whole approach incorporates community input. I would think that could only enhance it, could only improve trust because you’re no longer so much on the outside parachuting in and taking something from the community and leaving; you’re actually involving up front and getting input and I think that can only help enhance the trust that you would hope is there already …	8. In engaged research, attention must be paid not only to risks, benefits, and autonomy of individual research participants, but risks, benefits, and autonomy as they relate to communities.
		9. Identification of potential participants should be informed by community and academic researcher expertise to ensure fair selection and scientific validity.
		10. The process of obtaining consent should be informed by community and academic researcher expertise to take into account cultural, historical, and social context.
		11. Communities should provide input as to what constitutes acceptable risks and benefits.
Accountability	Academic: “Researchers have not traditionally gotten results back to the community of which they were obtained they…they went off and published in professional journals and that was their target for the results of research and now more and more there’s both an expectation, and certainly in community engaged research … If the community gives you this, they get something back for it. And so how to communicate results to people what does that mean, what does that look like, how long does that go on for.”	12. Researchers and communities are accountable for their presence and impact.
		13. Findings and data should be accessible to every stakeholder in order to increase dissemination of results and support sustainability.
		14. Community and academic researchers should aim for either the sustainability, responsible closure, or transition of projects.
		15. Community and academic researchers should commit to building and maintaining relationships over time.

#### Vision of equitable and just research

Vision of equitable and just research is the central domain, in that stakeholders heavily emphasized its overarching importance in the ethical review and conduct of research. Our findings raised the question of what is considered *just* in the context of engaged scholarship. From our findings, there is no single shared definition of justice. Instead, to ensure a shared set of values and expectations, community and academic partners should define justice and its implications for the research design, conduct, and communication. The vision of equitable and just research statements (#1–4) provide guidance in determining what justice means for a particular partnership and project. For example, in a given project, partners design, conduct, and communication of research need to be responsive to specific historic and/or ongoing points of injustice experienced by communities.

We found that stakeholders frame questions of justice at the community level. The Belmont Report [9] framed the topic by asking, “Who ought to receive the benefits of research and bear its burdens?” Based on our findings, the stakeholder-driven question becomes, “How, when designing, conducting, and communicating health research, do we promote justice for communities?” This is especially important when considering that some communities may be considered vulnerable in the research process *and* potentially have the most to gain from advances in research for social, economic, and/or historical reasons. Therefore, engaged scholarship ethics should emphasize research that focuses both on what communities care about and also finds linkages between community and research priorities.

With an emphasis on community throughout engaged scholarship partnerships, partners need to make a clear and explicit effort to ensure that communities are left better off than when the research began. In our findings, improving the community is not limited to the health outcomes of a given study to be reported in a publication. Rather, improvement to the community is a broad call to achieve equity. Community improvement takes on many forms, including equitable division of resources between academic and nonacademic entities to complete the proposed research, as well as capacity building within communities and stakeholder organizations to carry out and manage aspects of the research and participate effectively as co-investigators.

#### Relationship dynamics

The goal of this domain is to address power hierarchies inherent in the social structures that lead to health inequalities. A key power hierarchy manifests in the historic relationships between academic institutions and communities that can shape the research process. Relationship dynamics statements (#5–7) provide recommendations to meet this goal. Our findings underline the fundamental importance of building and sustaining relationships across time, including before project initiation. Building and sustaining relationships in community-engaged research requires that stakeholders are a part of all phases of the research continuum—identifying research questions, planning, implementing, and disseminating findings. The intended result is an increased level of transparency built on openness and honesty, where relationships are characterized by mutual trust, respect, benefit, and shared power and knowledge.

Many successful and long-standing research collaborations have explicitly built partnership capacity by developing shared language, systems, structures, and capacities that identify historical and contemporary oppressions that influence partnerships and the process of research [24]. This shared understanding in turn allows research teams to explicitly address the goal of action for health equity. Partnership capacity building efforts often include understanding how racism, elitism, and other forms of discrimination are experienced in the partnership and may be a microcosm of larger societal forms of oppression.

#### Community-informed risks and benefits

The conduct of research in a historically disadvantaged community may involve risks and benefits that are difficult for research funders, investigators, or ethics committees to anticipate. The community-informed risks and benefits statements (#8–11) further clarify that community engagement is necessary across many areas of ethical review, including fair selection of potential participants, informed consent procedures, autonomy of individuals, and risks/benefits to individuals and community. Throughout stakeholder engagement, we found that there was a dual emphasis on the community itself and on individuals in the community. To draw on their unique perspective and knowledge, stakeholders engaged in the research must be part of the assessment of risks and benefits.

Our emphasis on community engagement in risks/benefits assessment is consistent with recent trends. In recognition of the limited scope of academic IRBs, there is growing interest in community review boards that consider topics that represent perspectives of stakeholders regarding risks and benefits of the proposed research [24, 25]. In contrast to university’s focus on the production of new knowledge, groups historically underserved by medicine and research (e.g. patients, stakeholders, community members) often take into account cultural, historical, and social contexts of the community, how research and medicine have impacted the community in previous studies, and broader expectations of benefits. Because these factors are not typically considered in academic ethics review, communities have developed innovative models for assessing community risk/benefit, including Community Review Processes [24, 25]. Community Review Processes are community-based and lead processes for research ethics review. Their role is to consider the extent to which proposed research benefits or harms the community’s norms and values and to serve as gatekeepers to minimize harm. The community-led review is especially important in light of the power dynamics at play with university IRBs. Holding IRB processes at a university reinforces the hierarchy of academic institutions having greater decision-making power compared to community organizations, despite the latter being more directly impacted by the implemented research. Regardless of the IRB setting—community or university, research partners should have a shared understanding of the research ethics, regulatory processes, and timelines associated with institutional approvals and reporting.

#### Accountability

All partners in the research process have the responsibility to ensure that the process and products of engaged scholarship adhere to its underlying principles. This includes the expectation that research will lead to actions to improve health, co-learning and capacity building, community rights to self-determination, mutual respect, and shared power [4, 7]. Though several authors have made important contributions to providing a framework to assess research related risks for communities and individuals [4, 6, 26], our findings extend the current calls to accountability. Notably, our findings suggest that there is mutual accountability for the potential societal benefits of research as part of the risk/benefit calculus. Outlined in the accountability statements (#12–15), these benefits include building research-related capacity, ensuring the availability and use of research findings and products by all partners to improve community health, and sustaining the research partnership, the intervention, or both.

Inherent in the conduct of engaged scholarship, we also realize research relationships may benefit all stakeholders by creating new social ties, insights, and experiences. To ensure effective working relationships—both now and in the future—all parties in engaged scholarship should play an active role in developing, maintaining, and, when necessary, respectfully ending research collaborations. The ending of research collaborations requires particular care, as our findings suggest that all partners have a commitment to building and maintaining relationships over time. Beyond the partnership, all research partners have responsibilities to individuals and communities that are part of the research process and, as noted above, to each other. In particular, fidelity to the principles of engaged approaches ensures a focus on action and translation to improve health as both academic and community researchers become the face of the research project in their communities and as research findings are disseminated in communities and peer reviewed literature.

#### Cross-cutting considerations

As indicated, the following cross-cutting considerations pervade all domains: (1) capacity building, (2) translation for improved health, (3) individual vs. group concerns: collective ethos, and (4) power/hierarchy. Regarding capacity building, stakeholders suggested that ethical conduct of engaged scholarship should take into consideration the potential for capacity building. The capacities are not necessarily linked to a project’s specific research activities. Rather, capacity building is meant to go beyond any single study and aim for community change.

Regarding translation for improved health, ethical conduct should take into consideration the potential of the project to improve the community’s health. In other words, engaged scholarship should not aim to yield data for data’s sake or to only support an academic institution’s objectives. Instead, research should be conceived, designed, and conducted to increase the likelihood that the projects yield sustainable programs with the ultimate goal of improved community health.

Regarding collective ethos, the ethical conduct of engaged scholarship considers the potential to benefit the individual research participants *and* the community. Among stakeholders, what research does or does not do for a community was a common concern. Paired with this concern, stakeholders observed that an exclusive focus on the welfare of individual participants has often been at the expense of addressing group-level concerns. Attention to the group brings into focus the historical dimension of community, including past policies/actions that are part of collective memory and connected to current health issues.

The final cross-cutting consideration is power/hiearchy. Stakeholders raised concerns that, though issues of power are pervasive, so is the tendency to neglect or gloss over issues of power. Across all domains, power dynamics shape engaged scholarship. For example, academic researchers in community engaged research are typically in positions of social, economic, or political power relative to the communities with whom they partner. As mentioned above, IRBs being housed at universities, as opposed to held within community settings, reinforces the relative power of academic institutions. Collaborative decision-making power is jeopardized when community partners are not routinely involved in review and regulation of research ethics. Stakeholders recommended that power dynamics need to be observed, named, and discussed, in order to help minimize negative impacts.

### Limitations

Using a robust, multi-staged approach, this framework emphasized stakeholder engagement to yield domains, cross-cutting considerations, and domain-specific recommendations. Our study could have been enhanced by using other rigorous techniques, such as conducting a systematic review as opposed to narrative review, soliciting additional case studies to ensure broad representation, additional recruitment efforts to ensure higher response rate for the Delphi survey, and utilizing stratified sampling for the Delphi survey. Nonetheless, the iterative, multi-staged process corrected for biases and other limitations by not using other techniques. We urge caution in unreflective application of the findings presented here. Additional evidence would further clarify and support recommendations for the ethical review and conduct of engaged scholarship. We suggest that the 15 statements could be adapted into a checklist with a ranking system. Future research could pilot test the checklists use in IRB processes or ongoing community-academic partnered research. In tandem, we recommend all parties engage in ongoing scrutiny to refine their own ethical practices and policies.

## Conclusion

We present a framework for the ethical review and conduct of engaged scholarship. By engaging stakeholders across various settings (academic researchers, community researchers, and ethicists,), this framework extends the current discourse on the ethics of engaged scholarship. Our goal is to offer a stakeholder-engaged, data-driven framework upon which policies and practices can be built. This framework and recommendation could be made a part of continuing education and schedule of procedures for IRB members, particularly those committees that are charged with the review of engaged research. We offer these recommendations for community advisory boards and patient representatives on research studies to frame ethical concerns in discussions with other research stakeholders. In addition, we see opportunities to integrate these recommendations in existing IRB standard operating procedures. Finally, we see this framework and recommendations included in institutional offerings of the responsible conduct of research to ensure that both researchers and research staff based at academic institutions prioritize the ethical priorities of engaged scholarship. We hope the framework will be useful to key players who may influence their institutional and organizational policies, researchers co-leading the next generation of public health and biomedical research, and community partners invested in co-creating a platform for equitable research.
